# Albuminuria Is Associated with Endothelial Dysfunction and Elevated Plasma Endothelin-1 in Sickle Cell Anemia

**DOI:** 10.1371/journal.pone.0162652

**Published:** 2016-09-26

**Authors:** Kenneth I. Ataga, Vimal K. Derebail, Melissa Caughey, Laila Elsherif, Jessica H. Shen, Susan K. Jones, Poulami Maitra, David M. Pollock, Jianwen Cai, David R. Archer, Alan L. Hinderliter

**Affiliations:** 1 Division of Hematology/Oncology, University of North Carolina, Chapel Hill, NC, United States of America; 2 Division of Nephrology and Hypertension, University of North Carolina, Chapel Hill, NC, United States of America; 3 Division of Cardiology, University of North Carolina, Chapel Hill, NC, United States of America; 4 Department of Biochemistry and Biophysics, University of North Carolina, Chapel Hill, NC, United States of America; 5 Department of Biostatistics, University of North Carolina, Chapel Hill, NC, United States of America; 6 Division of Nephrology, The University of Alabama at Birmingham, Birmingham, AL, United States of America; 7 Department of Pediatrics, Emory University, Atlanta, GA, United States of America; Université Claude Bernard Lyon 1, FRANCE

## Abstract

**Background:**

The pathogenesis of albuminuria in SCD remains incompletely understood. We evaluated the association of albuminuria with measures of endothelial function, and explored associations of both albuminuria and measures of endothelial function with selected biological variables (vascular endothelial growth factor [VEGF], endothelin-1 [ET-1], soluble fms-like tyrosine kinase-1 [sFLT-1], soluble vascular cell adhesion molecule-1 [soluble VCAM-1] and plasma hemoglobin).

**Methods:**

Spot urine measurements for albumin-creatinine ratio (UACR) and 24-hour urine protein were obtained. Endothelial function was assessed using brachial artery ultrasound with measurements of flow-mediated dilation (FMD), nitroglycerin-mediated dilation (NTMD) and hyperemic velocity.

**Results:**

Twenty three subjects with varying degrees of albuminuria were evaluated. UACR was significantly correlated with FMD (ρ = -0.45, p = 0.031). In univariate analysis, UACR was correlated with VEGF (ρ = -0.49; 95% CI: -0.75 –-0.1, p = 0.015), plasma hemoglobin (ρ = 0.50; 95% CI: 0.11–0.75, p = 0.013) and ET-1 (ρ = 0.40; 95% CI: -0.03–0.69, p = 0.06). Multivariable analysis showed significant associations of ET-1 (estimate: 455.1 [SE: 198.3], p = 0.02), VEGF (estimate: -1.1 [SE: 0.53], p = 0.04) and sFLT-1 (estimate: -1.14 [SE: 0.49], p = 0.02) with UACR. Only ET-1 (estimate: -8.03 [SE: 3.87], p = 0.04) was significantly associated with FMD in multivariable analyses. Finally, UACR was correlated with both 24-hour urine protein (ρ = 0.90, p < 0.0001) and urine aliquots for albumin-creatinine ratio obtained from the 24-hour urine collection (ρ = 0.97, p < 0.0001).

**Conclusion:**

This study provides more definitive evidence for the association of albuminuria with endothelial dysfunction in SCD. Elevated circulating levels of ET-1 may contribute to SCD-related glomerulopathy by mediating endothelial dysfunction.

## Introduction

The survival of patients with sickle cell disease (SCD) into adulthood is associated with an increased incidence of organ dysfunction. It is well recognized that SCD is characterized by a vasculopathy which is thought to result in multiple clinical complications including ischemic stroke, pulmonary hypertension, autosplenectomy, priapism, and chronic kidney disease [1] 2009;9:271–292.

The term “sickle vasculopathy” has been used to describe a generalized form of endothelial dysfunction [2]. Similar to patients with coronary artery disease, atherosclerosis and its risk factors, patients with SCD exhibit impaired endothelium-dependent vascular reactivity, measured as flow-mediated dilation (FMD) of the brachial artery [3–5] or as the increase in flow induced by infusion of acetylcholine [6]. Multiple studies show associations of both albuminuria and elevated serum creatinine levels with echocardiography-derived tricuspid regurgitant jet velocity (TRV) and other vasculopathic complications in SCD [7–10] suggesting a shared pathophysiology. Despite the compelling evidence of endothelial dysfunction in SCD, its role in the pathophysiology of SCD-related complications remains poorly defined.

Our primary hypothesis is that endothelial dysfunction is an important contributor to the pathophysiology of albuminuria in SCD. The present study evaluates the association of measures of endothelial function, assessed non-invasively by ultrasound imaging of the brachial artery, with albuminuria in patients with SCD. In addition, we explored the association of multiple biological variables with albuminuria, as well as the association of these variables with measures of endothelial function.

## Patients and Methods

### Patients and Study Design

Patients with HbSS or HbSβ^0^ thalassemia and varying degrees of albuminuria, normal albuminuria (formerly called normoalbuminuria [urine albumin-creatinine ratio {UACR} < 30 mg/g]), moderately increased albuminuria (formerly called microalbuminuria [UACR: 30–299 mg/g]) and severely increased albuminuria (formerly called macroalbuminuria [UACR: ≥ 300 mg/g]), were recruited from the Sickle Cell Clinic at the University of North Carolina (UNC) at Chapel Hill. Spot urine samples were obtained for albumin-creatinine ratio over 2–3 visits in a three to six month period during the non-crisis “steady state.” The UACR obtained in the final spot urine collection was used to ascertain the degree of albuminuria. A 24-hour urine collection to assess protein and creatinine clearance was obtained at the final visit. Study subjects were evaluated in the non-crisis, “steady state” with no acute pain episodes requiring medical contact during the preceding 4 weeks; had normal baseline prothrombin and activated partial thromboplastin times; had acceptable hematologic, hepatic, neurologic, cardiovascular and endocrine function; were able to understand the study requirements and willing to give informed consent; and individuals receiving hydroxyurea or renin-angiotensin-aldosterone system blocking agents (such as angiotensin converting enzyme inhibitors or angiotensin receptor blockers) were required to be on stable doses for at least 3 months. Patients were excluded if they were pregnant; had a history of poorly controlled hypertension; had a history of diabetes mellitus; had a history of hypercholesterolemia; were on treatment with a statin; had chronic daily use of non-steroidal anti-inflammatory drugs; were breastfeeding; were on a chronic transfusion program; had conditions that increase the risk associated with or complicate interpretation of FMD and nitroglycerin-mediated dilation (NTMD) measurements (including known upper extremity vascular obstruction, severe aortic stenosis, hypertrophic obstructive cardiomyopathy, systolic blood pressure < 90 mmHg, treatment with a long-acting form of nitroglycerin, use of a phosphodiesterase-5 inhibitor, and allergy to nitroglycerin); or had ingested any investigational drugs within the preceding 4 weeks. The study was approved by the Institutional Review Board at UNC, Chapel Hill and all subjects gave written informed consent to participate in accordance with the Declaration of Helsinki.

### Assessment of Endothelial function

Vascular endothelial function was assessed by measuring endothelium-dependent (flow-mediated) and endothelium-independent (nitroglycerin-mediated) dilation of the brachial artery as described previously [11,12]. In addition, hyperemic velocity, a reflection of small vessel reactivity, was measured. Briefly, subjects reported to the research ultrasound laboratory in the morning after fasting (patients were allowed to drink water) and after having abstained from vigorous exercise, tobacco, and caffeine for at least 6 hours. Vasoactive medications were held for at least 4 half-lives prior to imaging. Ultrasound images of the right brachial artery were acquired proximal to the antecubital fossa, using a Philips Epiq 7 with a 12 MHz linear-array transducer (Koninklijke Philips N.V., Amsterdam, Netherlands). The first set of baseline images were obtained after 10 minutes of supine rest. Reactive hyperemia was induced by inflating a pneumatic occlusion cuff placed around the upper forearm to a suprasystolic pressure (~ 200 mmHg) for 5 minutes, and then deflating the cuff. Pulsed wave Doppler tracings representing arterial flow were acquired immediately after cuff release. Images of the artery were then recorded for two minutes. After 10 minutes of rest, a second set of baseline images were acquired. Sublingual nitroglycerin (0.4 mg) was then administered with acquisition of ultrasound images for the subsequent 5 minutes. Measurements were performed using Vascular Tools (Medical Imaging Applications, Coralville, IA). Arterial diameter was measured from the lumen-intimal interfaces of the proximal and distal arterial walls. Data from at least ten end-diastolic frames were averaged for each baseline measurement and from at least three frames at maximum dilation during reactive hyperemia and following administration of nitroglycerin. FMD was calculated as the % change in arterial diameter in response to reactive hyperemia, and NTMD was quantified as the % diameter change following administration of nitroglycerin. The hyperemic velocity was calculated as the planimetered time-velocity integral of the first complete velocity envelope obtained after cuff release.

### Laboratory Analysis

Blood samples were obtained via venipuncture and drawn into citrate- and heparin-containing tubes. The plasma samples were aliquoted and frozen immediately at -80°C for subsequent analysis. Quantification of vascular endothelial growth factor (VEGF), endothelin-1 (ET-1), soluble fms-like tyrosine kinase-1 (sFLT-1, also referred to as soluble vascular endothelial growth factor receptor-1 [VEGFR-1]) and human soluble vascular cell adhesion molecule-1 (soluble VCAM-1) were accomplished using commercially available ELISA kits (R&D systems, Minneapolis, MN [human Quantiglo catalog number QET00B for ET-1 analysis]). Samples were assayed in duplicate and according to manufacturer's instructions. The quantification of free plasma hemoglobin was accomplished using a laboratory developed test (LDT, Core Laboratory, McLendon Clinical Laboratories, UNC, Chapel Hill) adapted to the Vitros 5600 chemistry platform (Ortho Clinical Diagnostics, Raritan NJ). The absorbance at 600 nm was subtracted from the absorbance at 575 nm and multiplied by a fixed calibration factor that took into account any performed dilutions. Routine laboratory studies, including complete blood counts, chemistries to assess renal function, liver function and measures of hemolysis, spot urine for albumin-creatinine ratio and 24-hour urine for protein were performed by the McClendon Clinical Laboratory at UNC Hospitals. Glomerular filtration rate (GFR) was estimated by an equation developed by the Chronic Kidney Disease Epidemiology (CKD-EPI) Collaboration [13]. Hemoglobin analysis was performed by high performance liquid chromatography to confirm the SCD diagnosis and ascertain fetal hemoglobin levels.

### Statistical analyses

Descriptive statistics were computed for the primary outcome variables and other covariates. Medians and the corresponding interquartile ranges are presented. Albuminuria was analyzed as both continuous and categorical variables for testing the primary hypothesis. For the primary hypothesis, the correlations between FMD, NTMD or hyperemic velocity and albuminuria were examined using Spearman’s rank correlation coefficient. The correlations between endothelial function measures and biological variables were also examined using Spearman’s rank correlation coefficient. Univariate and multivariable regression analyses were conducted to investigate the association of selected biological variables with albuminuria and FMD. Because the distribution of albuminuria and FMD were skewed, the bootstrap method was used with 10,000 replications to obtain the p value and 95% confidence interval [14]. A backward selection procedure was used for variable selection. The deletion criterion was based on a p-value > 0.05 and the variable with the largest p value was deleted first at each step. The final model included only those variables which were statistically significant at 0.05 level. Reported p-values are for individual tests, unadjusted for multiple comparisons because of the exploratory nature of this study. All analyses were performed using SAS statistical software v9.4 (SAS Institute, Cary, NC).

## Results

### Demographic and Laboratory Characteristics

Twenty three patients with HbSS (female: 13), and with a median age of 42 years (range: 25–67 years), were enrolled. Normal albuminuria was present in 7 subjects, moderately increased albuminuria in 9 subjects, and severely increased albuminuria in 7 subjects. Consistent with their definitions, patients with severely increased albuminuria had higher median UACR than those with moderately increased albuminuria and normal albuminuria. Patients with severely increased albuminuria appeared to be older, and had significantly higher systolic and diastolic blood pressures than those patients with moderately increased or normal albuminuria (Table 1). In addition, patients with severely increased albuminuria and moderately increased albuminuria had higher levels of serum creatinine than those with normal albuminuria.

**Table 1:** Baseline Demographic and Laboratory data.

Variable	**Normal Albuminuria (N = 7)	**Moderately Increased Albuminuria (N = 9)	**Severely Increased Albuminuria (N = 7)	P Value
Age (years)	37 (31–46)	40 (34–48)	50 (40–57)	0.24
Gender (F)	6 (85.7)	4 (44.4)	3 (42.9)	0.23
Weight (kg)	62.3 (58.7–95.8)	66.1 (60.1–68.3)	76.7 (70.8–78.2)	0.28
Height (cm)	166.1 (163.5–172.5)	171.4 (166.1–176.8)	168.0 (163.5–172.0)	0.42
Systolic Blood Pressure (mm Hg)	113 (95–121)	120 (117–145)	138 (117–151)	0.045
Diastolic Blood Pressure (mm Hg)	58 (52–66)	63 (56–67)	81 (63–83)	0.035
On Hydroxyurea (yes)	5 (71.4)	7 (77.8)	6 (85.7)	1.00
On renin-angiotensin-aldosterone system blocking agent (yes)	0 (0)	2 (22.2)	2 (28.6)	0.5
White Blood Cell count (×10^9^/L)	6.3 (5.0–8.6)	7.0 (5.8–10.6)	8.1 (4.8–11.2)	0.7
Hemoglobin (g/dL)	9.4 (8.7–10.4)	8.8 (7.5–9.2)	8.8 (6.8–15.2)	0.57
Platelet Count (×10^9^/L)	339.0 (302.0–637.0)	288.0 (224.0–374.0)	356.0 (253.0–445.0)	0.33
Reticulocyte Count (%)	5.1 (2.5–8.9)	9.9 (5.0–11.8)	6.0 (3.4–9.2)	0.11
Fetal Hemoglobin (%)	13.5 (2.5–24.0)	6.3 (5.4–16.1)	13.0 (8.0–9.5)	0.64
Creatinine (mg/dL)	0.56 (0.53–0.58)	0.69 (0.64–1.10)	0.7 (0.52–1.27)	0.044
*Estimated Glomerular Filtrate Rate (mL/min/1.73_m^2^)	129.8 (120.0–152.6)	113.1 (68.8–147.0)	127.4 (67.8–137.5)	0.19
Lactate Dehydrogenase (U/L)	733.0 (573.0–1093.0)	1067.5 (770.5–1389.0)	907.0 (759.0–998.0)	0.18
Total Bilirubin (mg/dL)	1.3 (0.9–6.3)	2.5 (1.75–3.5)	2.6 (2.1–4.2)	0.71
Direct Bilirubin (mg/dL)	0.09 (0.09–0.20)	0.35 (0.1–0.6)	0.3 (0.2–0.5)	0.069
Indirect Bilirubin (mg/dL)	1.21 (0.60–6.21)	2.40 (1.15–3.1)	2.3 (1.6–4.1)	0.87
Urine Albumin-Creatinine Ratio (mcg/mg)	9.42 (3.80–11.5)	95.8 (48.6–142.7)	518.9 (398.6–706.7)	< 0.0001

* Glomerular filtration rate estimate by CKD-EPI equation

** Medians (with Interquartile ranges) or Numbers (%)

### Urine Albumin-Creatinine Ratio is Associated with Flow Mediated Dilation

**Fig 1:**
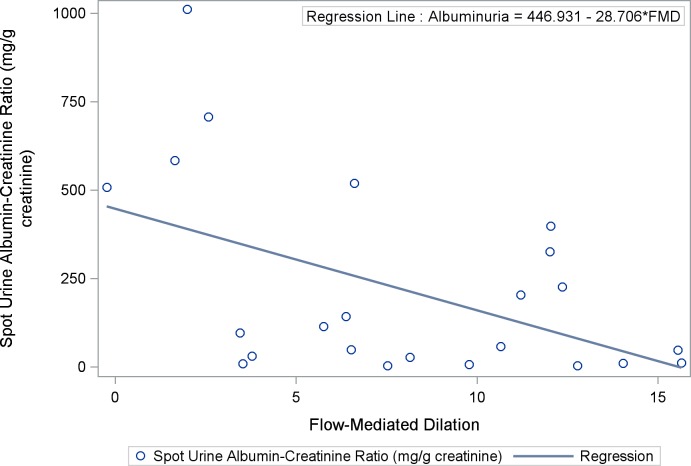
Scatter Plot and Regression Line Between Flow-Mediated Dilation and Albuminuria in Sickle Cell Anemia: Urine albumin-creatinine ratio is significantly correlated with FMD (ρ = -0.45; 95% CI: -0.72 –-0.04, p = 0.031).

**Fig 2:**
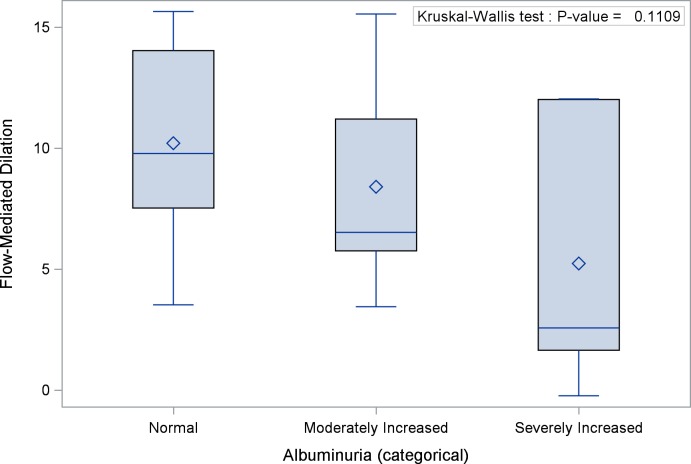
Distribution of Flow-Mediated Dilation by Albuminuria Categories: FMD is lowest in patients with severely increased albuminuria compared with those with moderately increased and normal albuminuria, although the difference is not statistically significant (p = 0.16).

UACR was negatively correlated with FMD (ρ = -0.45; 95% confidence interval [CI]: -0.72 –-0.04, p = 0.031) (Fig 1). In addition, a linear regression model showed that for every 1% increase in FMD, UACR decreased by 28.7 mcg/mg (p = 0.0076). Although FMD appeared to be lowest in patients with severely increased albuminuria, no significant difference was observed when FMD was evaluated in the 3 albuminuria categories (p = 0.16) (Fig 2). In a multinomial logistic regression model, the odds of severely increased albuminuria appeared to be reduced by 24% (odds ratio [OR]: 0.76; 95% CI: 0.57–1.01) for every 1% increase in FMD, while the odds of moderate albuminuria appeared to be reduced by 9% (OR: 0.91; 95% CI: 0.72–1.45). There was a trend towards an association between FMD and UACR after controlling for the baseline arterial diameter, although this was not statistically significant (p = 0.1). No significant correlations were observed between UACR and NTMD (ρ = -0.32 95% CI: -0.67–0.14, p = 0.15) or between UACR and hyperemic velocity (ρ = 0.08; 95% CI: -0.37–0.51, p = 0.73). In addition, neither NTMD nor hyperemic velocity was significantly associated with albuminuria when it was assessed as a categorical variable. The values of the measures of endothelial function based on albuminuria categories are shown in Table 2.

**Table 2:** Values of Measures of Endothelial Function Based on Albuminuria Category.

Variable	*Normal Albuminuria (N = 7)	*Moderately Increased Albuminuria (ACEi/ARB—No) (N = 7)	*Moderately Increased Albuminuria (ACEi/ARB—Yes) (N = 2)	*Severely Increased Albuminuria (ACEi/ARB—No) (N = 5)	*Severely Increased Albuminuria (ACEi/ARB—Yes) (N = 2)
Flow-Mediated Dilation (%)	9.78 (7.53, 14.04)	10.65 (5.76–12.4)	4.91 (3.45–6.38)	6.61 (2.57–12.0)	1.82 (1.65–1.99)
Nitroglycerin-mediated Dilation (%)	30.6 (17.6, 33.8)	23.1 (20.07–32.17)	23.96 (19.8–28.1)	30.3 (17–31.8)	13.3 (11.3–15.3)
Hyperemic Index (cm/s)	7.50 (6.0, 24.0)	89 (88–124)	11 (5–7)	15 (7–20)	46 (16–76)

* Medians and Interquartile Ranges

ACEi/ARB–Angiotensin Converting Enzyme Inhibitor/Angiotensin Receptor Antagonist

### Association of Urine Albumin-Creatinine Ratio with Biological Variables

The levels of evaluated biological variables based on the albuminuria categories are shown in Table 3. UACR was significantly correlated with VEGF (ρ = -0.49; 95% CI: -0.75 –-0.1, p = 0.015) and free plasma hemoglobin (ρ = 0.50; 95% CI: 0.10–0.75, p = 0.013), with a trend towards a significant correlation with plasma ET-1 (ρ = 0.40; 95% CI: -0.03–0.69, p = 0.06) (Table 4). However, no significant correlations were observed between UACR and sFLT-1 (ρ = 0.02; 95% CI: -0.40–0.43, p = 0.94) or soluble VCAM-1 (ρ = 0.08; 95% CI: -0.36–0.48, p = 0.73) in univariate analyses. No significant correlations were observed between UACR and hemoglobin, fetal hemoglobin, lactate dehydrogenase or indirect bilirubin.

**Table 3:** Levels of Biological Variables Based on Albuminuria Category.

Variable	*Normal Albuminuria (N = 7)	*Moderately Increased Albuminuria (ACEi/ARB—No) (N = 7)	*Moderately Increased Albuminuria (ACEi/ARB—Yes) (N = 2)	*Severely Increased Albuminuria (ACEi/ARB—No) (N = 5)	*Severely Increased Albuminuria (ACEi/ARB—Yes) (N = 2)
Endothelin-1 (pg/mL)	0.44 (0.28–0.69)	0.80 (0.58–0.90)	0.65 (0.62–0.68)	0.65 (0.49–0.74)	0.84 (0.67–1.02)
VEGF (pg/mL)	100.2 (79.1–290.8)	51.8 (38.1–138.9)	125.5 (50.7–200.3)	50.16 (47.6–59.7)	61.42 (36.5–86.4)
Log(Plasma Hemoglobin)	1.61 (1.39–2.49)	3.37 (2.30, 3.78)	2.22 (1.79–2.64)	2.94 (2.89–3.33)	2.59 (2.40–2.77)
sFLT-1 (pg/mL)	131.2 (101.1–181.2)	220.9 (129.6–309.3)	197.1 (183.7–210.5)	156.16 (124.8–182.6)	89.48 (65.5–113.5)
Soluble VCAM-1 (ng/mL)	1460.3 (569.5–1871.3)	1153.23 (829.5–2389.0)	1660.55 (1054.3–2266.9)	1428.78 (941.3–1966.4)	1427.20 (1408–1446.4)

* Medians and Interquartile Ranges

VEGF–Vascular Endothelial Growth Factor; sFLT-1 –Soluble FMS-Like Tyrosine Kinase-1; Soluble VCAM-1 –Soluble Vascular Cell Adhesion Molecule-1

**Table 4:** Spearman Correlation of Urine Albumin-Creatinine Ratio with Biological Variables.

Variable	ρ (95% Confidence Interval)	P Value
VEGF	-0.49 (-0.75 –-0.1)	0.015
Endothelin-1	0.40 (-0.03–0.69)	0.06
sFLT-1	0.02 (-0.40–0.43)	0.94
Soluble VCAM-1	0.08 (-0.36–0.48)	0.73
Log (Plasma Hemoglobin)	0.50 (0.11–0.75)	0.013
Lactate Dehydrogenase	-0.19 (-0.25–0.57)	0.38
Indirect Bilirubin	0.12 (-0.32–0.51)	0.60
Fetal Hemoglobin	-0.11 (-0.52–0.34)	0.60
Hemoglobin	-0.16 (-0.53–0.27)	0.47

VEGF–Vascular Endothelial Growth Factor; sFLT-1 –Soluble FMS-Like Tyrosine Kinase-1; Soluble VCAM-1 –Soluble Vascular Cell Adhesion Molecule-1

### Association of Biological Variables with Flow Mediated Dilation

There were modest correlations between FMD and ET-1 (ρ = -0.39; 95% CI: -0.69–0.04, p = 0.07), FMD and VEGF (ρ = 0.35; 95% CI: -0.072–0.67, p = 0.09), and FMD and soluble VCAM-1 (ρ = -0.32; 95% CI: -0.66–0.11, p = 0.13), although these were not statistically significant. No significant correlations were observed between FMD and either sFLT-1 (ρ = 0.09; 95% CI: -0.34–0.48, p = 0.69), or plasma hemoglobin (ρ = -0.11; 95% CI: -0.50–0.32, p = 0.62) in univariate analyses. In addition, there were no significant correlations between FMD and hemoglobin, fetal hemoglobin, lactate dehydrogenase, indirect bilirubin, triglyceride, LDL-cholesterol, HDL-cholesterol or non-HDL-cholesterol (Table 5).

**Table 5:** Spearman Correlation of Flow Mediated Dilation with Biological Variables.

Variable	ρ (95% Confidence Interval)	P value
VEGF	0.35 (-0.072–0.67)	0.09
Endothelin-1	-0.39 (-0.69–0.04)	0.07
sFLT-1	0.09 (-0.34–0.48)	0.69
Soluble VCAM-1	-0.32 (-0.66–0.11)	0.13
Log (Plasma Hemoglobin)	-0.11 (-0.50–0.32)	0.62
Triglyceride	-0.08 (-0.47–0.35)	0.73
LDL-Cholesterol	0.02 (-0.39–0.43)	0.92
HDL-Cholesterol	-0.28 (-0.62–0.15)	0.18
Non-HDL-Cholesterol	0.11 (-0.32–0.50)	0.63
Hemoglobin	0.25 (-0.18–0.60)	0.25
Lactate Dehydrogenase	-0.24 (-0.60–0.21)	0.28
Indirect Bilirubin	-0.20 (-0.50–0.24)	0.36
Fetal Hemoglobin	0.25 (-0.20–0.62)	0.26

VEGF–Vascular Endothelial Growth Factor; sFLT-1 –Soluble FMS-Like Tyrosine Kinase-1; Soluble VCAM-1 –Soluble Vascular Cell Adhesion Molecule-1

### Multivariable Analyses

Multivariable analysis was conducted to investigate selected biological factors that could be associated with UACR in the study subjects. A backward variable selection procedure was used for variable selection. The initial model included ET-1, VEGF, sFLT-1, soluble VCAM-1, log(plasma hemoglobin), lactate dehydrogenase, indirect bilirubin, hemoglobin, and fetal hemoglobin. In the final model, using only significant covariates after the model selection, ET-1 (estimate: 455.1 [SE: 198.3], p = 0.02), VEGF (estimate: -1.1 [SE: 0.53], p = 0.04) and sFLT-1 (estimate: -1.14 [SE: 0.49], p = 0.02), were significantly associated with UACR. This means, for example, that we expect an increase in UACR by 455.1 mcg/mg for every 1 pg/mL increase in plasma ET-1, given the same levels of VEGF and sFLT-1.

Similarly, multivariable analysis was conducted to investigate selected biological factors that could be associated with FMD in the study patients. The initial model included ET-1, VEGF, sFLT-1, soluble VCAM, log(plasma hemoglobin), lactate dehydrogenase, indirect bilirubin, hemoglobin, fetal hemoglobin, triglyceride, LDL-cholesterol, HDL-cholesterol, and non-HDL-cholesterol. In the final model, using only significant covariates after the model selection, only ET-1 (estimate: -8.03 [SE: 3.87], p = 0.04) was significantly associated with FMD. This means that we expect a decrease in FMD by approximately 8% for every 1 pg/mL increase in plasma ET-1.

### Spot Urine Albumin-Creatinine Ratio Correlates with 24-Hour Urine Protein

**Fig 3:**
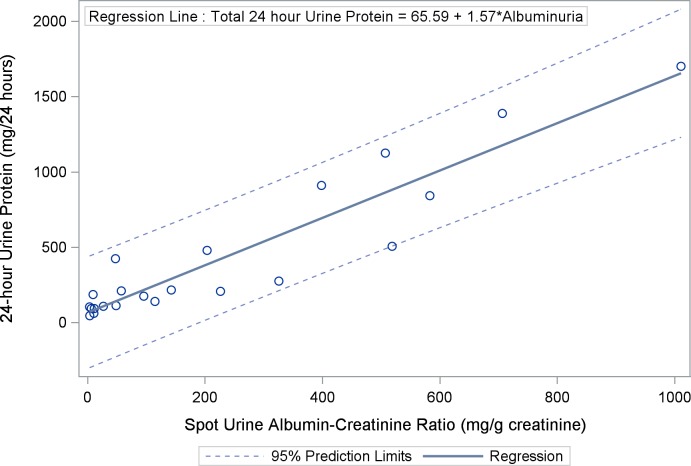
Scatter Plot and Regression Line Between Spot Urine Albumin-Creatinine Ratio and 24-Hour Urine Protein: Urine albumin-creatinine ratio is strongly correlated with total urinary protein assessed by 24-hour urine collection (ρ = 0.90; 95% confidence interval [CI]: 0.75–0.95, p < 0.0001).

**Fig 4:**
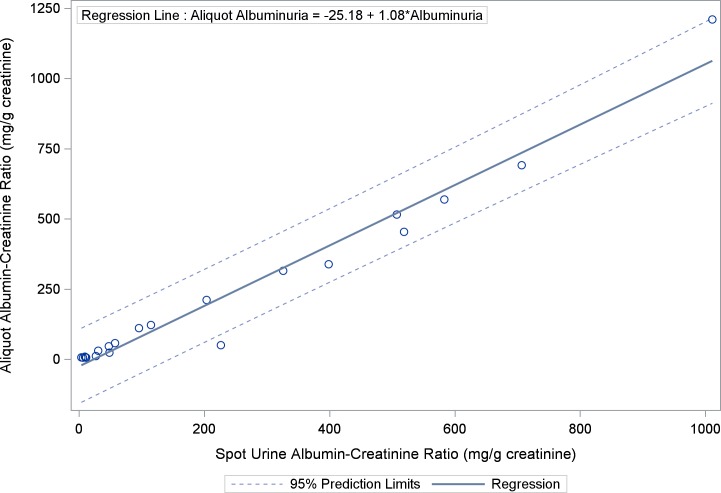
Scatter Plot and Regression Line Between Spot Urine Albumin-Creatinine Ratio and Urine Aliquot for Albumin-Creatinine Ratio: Urine albumin-creatinine ratio assessed by spot albumin-creatinine ratio is strongly correlated with urine aliquots for albumin-creatinine ratio obtained from the 24-hour urine collection (ρ = 0.97; 95% CI: 0.92–0.99, p < 0.0001).

As UACR, assessed by spot urine collections, was the primary variable of interest in this study, we evaluated the correlation between this variable and 24-hour urine protein collection, the gold standard for assessment of proteinuria. UACR was strongly correlated with total urinary protein assessed by 24-hour urine collection (ρ = 0.90; 95% CI: 0.75–0.95, p < 0.0001) (Fig 3). In addition, UACR assessed by spot albumin-creatinine ratio was strongly correlated with urine aliquots for albumin-creatinine ratio obtained from the 24-hour urine collection (ρ = 0.97; 95% CI: 0.92–0.99, p < 0.0001) (Fig 4).

## Discussion

Albuminuria, the most common clinical manifestation of glomerular damage, is highly prevalent in patients with SCD. The prevalence of albuminuria increases with age, with estimates of between 4.5 and 26% in patients up to 21 years, and between 26 and 68% in older patients [15]. The pathophysiology of sickle cell glomerulopathy is incompletely understood, with possible contributions from glomerular hypertension, hyperfiltration and increases in oxidative stress [15]. There are conflicting data on the association of hemolysis (and consequent decreased nitric oxide [NO] bioavailability) with albuminuria in patients with SCD [15]. We have previously suggested a contribution of endothelial dysfunction to the pathophysiology of SCD-related glomerulopathy based on elevated levels of sFLT-1 and soluble VCAM-1 in patients with albuminuria [10]. In the present study, we find that UACR is negatively correlated with FMD, a measure of endothelium-dependent dilation of the brachial artery. This is the first physiologic measure to confirm the association of albuminuria with endothelial dysfunction in SCD and provides more evidence to support the hypothesis that endothelial dysfunction contributes to the pathophysiology of albuminuria in this condition. Echocardiography-derived TRV was recently reported to be inversely correlated with FMD in patients with SCD [16], although none of the patients obtained right heart catheterizations to confirm the presence of pulmonary hypertension.

The significant association of plasma ET-1 with UACR in the multivariable analysis suggests a role for ET-1 in the pathogenesis of albuminuria in SCD. ET-1, an endothelium-derived peptide, is a potent vasoconstrictor which is released in response to hypoxia, hemin, angiotensin II, sheer stress, thrombin activation and inflammatory cytokines [17]. It has been implicated in the pathogenesis and progression of diabetic kidney disease [18], a condition similar to SCD-related glomerulopathy. Previous studies in patients with SCD have reported elevated plasma levels of ET-1 [19,20], and a correlation of urinary ET-1 with albuminuria [20]. Furthermore, studies in transgenic sickle mice have demonstrated a contribution of ET-1 to progressive kidney injury via the ET_A_ receptor [17,21]. With our finding that plasma ET-1 is inversely associated with FMD, ET-1 may contribute to the pathogenesis of albuminuria in SCD by causing endothelial dysfunction. ET-1 appears to mediate endothelial dysfunction by reducing NO bioavailability both through its interference with the expression and activity of endothelial NO synthase [22], and the formation of reactive oxygen species [23–25]. With the possibility that ET-1 contributes to the pathogenesis of SCD glomerulopathy, antagonism of ET-1 could represent a novel therapeutic pathway. A study of the effect of the selective ET_A_ receptor antagonist, ambrisentan, to evaluate its safety and effect on albuminuria in patients with SCD is ongoing (www.clinicaltrials.gov identifier: NCT02712346).

A significant negative association was observed between UACR and VEGF. This is consistent with a previous report which showed that higher VEGF concentrations are associated with decreased odds of elevated TRV in children and young adults with SCD [26]. Podocytes (epithelial cells), attached to the glomerular basement membrane by discrete foot processes, both generate and respond to several angiogenic growth factors, including vascular endothelial growth factor A (VEGF-A). VEGF-A is important for maintaining the integrity of the glomerular filtration barrier and serves as a survival factor for the podocyte [27] by signaling through the VEGF-Receptor-2 (VEGFR2) which is abundantly expressed in the glomerular endothelium. Both excess amounts and a deficiency in the development or maturity of VEGF-A in the podocyte cause glomerular damage [28,29]. Excess VEGF-A induces endothelial growth and swelling (referred to as endotheliosis), while inadequate VEGF-A release causes endothelial damage and apoptosis leading to glomerulosclerosis [30]. VEGF-A levels and signaling must be closely regulated to maintain healthy glomerular structure and function [31]. Glomerular levels of VEGF-A may be elevated [32,33] or reduced [34] in diabetic kidney disease, with elevated levels observed early in the disease followed by decreased levels with disease progression. The negative association of UACR with sFLT-1 in the multivariable analysis was unexpected. We have previously reported an association of sFLT-1 with albuminuria in patients with SCD [10]. By antagonizing VEGF action, increased levels of sFLT-1 is reported to produce endothelial dysfunction and has been linked with proteinuria in preeclampsia [35].

We observed a significant correlation between UACR and plasma hemoglobin in univariate analysis, but not in the multivariable analysis. Although the published data on the association of albuminuria with markers of hemolysis in patients with SCD are conflicting [15], recent data show an association of hemoglobinuria with progression of CKD and albuminuria [36]. An association has been reported between APOL1 G1/G2 with chronic kidney disease in SCD, possibly through an increased risk of hemoglobinuria [37]. Furthermore, HMOX1 variants are associated with CKD, possibly through reduced protection of the kidney from hemoglobin-mediated toxicity [37]. In addition to the direct renal toxicity of free hemoglobin, chronic depletion of NO due to scavenging by cell-free hemoglobin may contribute to the pathogenesis of sickle cell glomerulopathy. Hemoglobin, released following lysis of red blood cells, is a potent NO scavenger, with even small amounts of cell-free plasma hemoglobin completely impairing NO signaling in the endothelium [38]. The level of cell-free plasma hemoglobin in SCD has been reported to correlate with an intrinsic resistance to NO signaling, based on impaired blood flow responses to infusions of NO donor medications in both humans and sickle mice [39,40]. Although, cell-free plasma hemoglobin has been recently shown to inversely correlate with FMD in patients with SCD [16], we did not observe a significant correlation between plasma hemoglobin and FMD, possibly due to the small size of this study.

Potential contributors to the pathobiology of the chronic vasculopathy and endothelial dysfunction of SCD include RBC sickling following hemoglobin S polymerization, systemic inflammation, upregulation of endothelial adhesion molecules, coagulation activation, decreased NO bioavailability, vascular instability with up-regulation of non-NO vasoregulators, disruption of normal endothelial signaling function, increased oxidative stress, vascular stasis and recurrent ischemia-reperfusion [1]. We now provide evidence for a contribution of ET-1 to the modulation of endothelial function in patients with SCD.

Although the gold standard for quantitative assessment of albuminuria is a 24-hour urine collection, many studies assessing albuminuria in SCD have been based on spot urine assessments of UACR. Urine albumin-creatinine ratio has not been validated in SCD. In the present study, we find a strong correlation between UACR and 24-hour urine protein collection, with an even stronger correlation between UACR and urine aliquots for albumin-creatinine ratio obtained from the 24-hour urine collection. Although proteinuria in SCD is mainly due to glomerular injury with subsequent albuminuria, patients are also known to have renal tubular dysfunction, with increased urinary concentrations of tubular proteins, such as beta-2-microglobulin and retinol-binding protein [41]. The presence of tubular proteins in 24-hour urine collections is a likely explanation for the slightly lower correlation of UACR with 24-hour urine protein in the present study. The very strong correlation observed between UACR and urine aliquots for albumin-creatinine ratio obtained from the 24-hour urine collection in this study provides support for the use of spot urine collections to evaluate albuminuria in adult patients with SCD.

Our study is limited by the small number of subjects in the albuminuria categories. As with all cross-sectional studies, this analysis demonstrates associations, but cannot prove causation. As most of the study subjects were on hydroxyurea, we did not evaluate the association of hydroxyurea use with endothelial function. Hydroxyurea has been reported to reduce albuminuria in patients with SCD [42,43], an effect that may be due, in part, to improved endothelial function. Hydroxyurea may improve endothelial function by decreasing hemolysis [44], decreasing the expression of VCAM-1 [45], decreasing the level of ET-1 [46], and by acting as an NO donor [47].

In summary, the present study, confirms the association of albuminuria with endothelial dysfunction in SCD. We also show that elevated plasma ET-1 levels may contribute to SCD-related glomerulopathy by mediating endothelial dysfunction. Studies to determine whether interventions that improve endothelial function can attenuate albuminuria in SCD are warranted.

## Supporting Information

S1 File Supporting information file is saved as S1_File.excel. (XLTX)
